# Rabies and Dog Bites Cases in Lagos State Nigeria: A Prevalence and Retrospective Studies (2006-2011)

**DOI:** 10.5539/gjhs.v6n1p107

**Published:** 2013-10-27

**Authors:** Sunday E. Hambolu, Asabe A. Dzikwi, Jacob K. P. Kwaga, Haruna M. Kazeem, Jarlath U. Umoh, Dupe A. Hambolu

**Affiliations:** 1 Department of Veterinary Public Health and Preventive Medicine, Faculty of Veterinary Medicine, Ahmadu Bello University, Zaria, Kaduna State, Nigeria; 2 Department of Veterinary Microbiology, Faculty of Veterinary Medicine, Ahmadu Bello University, Zaria, Kaduna State, Nigeria; 3 Federal Department of Livestock and Pest Control Services, Federal Ministry of Agriculture and Water Resources, Abuja, Nigeria

**Keywords:** bites, brain, dogs, rabies, retrospective, Lagos, Nigeria

## Abstract

This study was carried out to determine the prevalence of rabies antigen in brain of dogs slaughtered for consumption and those that died in veterinary clinics as well as to obtain a 6-year retrospective data on dog bites/suspected dog rabies cases in Lagos State. Dog brain samples were collected from dog slaughter slabs and veterinary clinics (for dogs that died in clinics) across the Lagos state while data for retrospective studies (2006-2011) of dog bite/suspected rabies cases were collected from public (government owned) and private veterinary clinics across the state. Out of the 444 brain samples collected and tested for presence of rabies antigen using the direct fluorescent antibody technique (DFAT) only 7 (1.58%) were positive for the rabies antigen. A total of 196 dog bites/suspected rabies cases were encountered between January 2006 and December, 2011 in the veterinary clinics with adults been the major (55.61%) victims. Majority (96.43%) of the offending dogs were not quarantined at the time of bite and only one out of the quarantined dogs died and was confirmed positive for rabies antigen. The result of this study indicates that rabies antigen is present among dogs slaughtered in Lagos State and may pose a threat to public health. Though, available records showed that provocation of dogs was the major cause of dog bites and both children and adults fell victim of dog bites, there was a poor record keeping practice in the veterinary clinics across the state.

## 1. Introduction

Rabies is a fatal viral disease that affects all warm blooded vertebrates (Zulu et al., 2009). It is one of the most important and widespread zoonotic diseases and a global dilemma (Blancou, 1988; WHO, 1991; WHO, 1998). The virus causing the disease belongs to genotype 1 of the genus *Lyssa virus* in the family *Rhabdoviridae*. The rabies virus genome consists of a single stranded, nonsegmented, negative sense RNA of approximately 12kb (Tordo et al., 1986). The **Lyssa virus** genus, within the **Rhabdoviridae** family, is subdivided into seven genotypes based on RNA sequencing (Bourhy et al., 1993a,b; 1999): Classical rabies virus (RABV, genotype 1 found worldwide), Lagos bat virus (genotype 2 found in Africa), Mokola virus (genotype 3 most common in Africa), Duvenhage virus (genotype 4 most common in Africa), European bat lyssavirus 1 (EBLV-1, genotype 5 most common in Europe), European bat lyssavirus 2 (EBLV-2, genotype 6 most common in Europe), and Australian bat lyssavirus (ABLV, genotype 7 most common in Australia) (Badrane et al., 2001; Gould et al., 1998). Also four putative viruses (Aravan, Khujand, Irkut and West Caucasian Bat Virus, Shimoni bat virus isolated in 2009) as well as Bokeloh bat lyssavirus (Freuling et al., 2011) and the Ikoma lyssavrus (Marston et al., 2012) were discovered in 2011 and 2012 respectively.

The disease agent is maintained and transmitted by a variety of different host species and widespread among domestic dogs (Ngoepe et al., 2009). The domestic dog *(Canis familiaris)* plays a pivotal role in rabies transmission, with 85-95% of human rabies cases being ascribed to dog bites (Tang et al., 2005) due to their close association with man (McKenzie et al., 1993). According to WHO report, ten million people are bitten by animals and considered for prophylaxis and treatment against rabies around the world yearly. Out of this number almost (55,000) people die from this disease annually (WHO, 2003). Besides the fact that dog bites are a serious health problem that can cause both physical and emotional trauma to victims and considerable cost to communities (Dwyer et al., 2007). It can serve as a route for the transmission of rabies to the victim especially if bitten by a rabied dog (Joo et al., 2011). Human rabies is practically 100% fatal (Mazigo et al., 2010) and is endemic among the dog population especially in Africa and Asia where domestic dogs serve as its major reservoir.

Several techniques such as; demonstration of Negri bodies by Sellers staining, Direct Fluorescent antibody test (FAT), Rapid Rabies Enzyme Immunodiagnosis (RREID), Latex agglutination Test, Virus isolation in new born mice, virus isolation in cell cultures, Immunoperoxidase test (IPT), Peroxidase and antiperoxidase test (PAP), Avidin-biotin test, Dipstick dot ELISA, Dot ELISA, Electron Microscopy and recently molecular methods (detection of rabies viral RNA by Dot and Slot Hybridization, *in situ* Hybridization and different types of RT-Polymerase Chain Reaction have been used to detect rabies antigen details of which have been published in many review articles (Fook et al., 2009; Madhusudana & Sukumaran, 2008; Woldehiwet, 2005). However, FAT is gold standard recommended by both WHO and OIE and the most widely used test for rabies diagnosis as it is highly sensitive, specificity, cheap and gives reliable results providing results within few hours in more than 95-99% of rabies cases (OIE, 2013). Despite the availability of vaccines to prevent this disease, it is still a significant public and veterinary health problem in many countries particularly in Asia and Africa (Meslin et al., 1994; Knobel et al., 2005; WHO, 1999) as a result of neglect (Warrell & Warrell, 1995), lack of accurate data on the true impact of the disease and lack of political commitment for its control (Knobel et al., 2005). More than 90% of all human deaths from rabies occur in the developing world (Ajayi et al., 2006). In Africa and Asia, an estimated 24,000-70,000 people die of rabies each year (Knobel et al., 2005) and the domestic dog is the main source of exposure and primary vector for this important human disease (Wandeler et al., 1993).

Results from various studies of brain samples from apparently healthy dogs in Nigeria have shown a prevalence of between 28-32% (Ajayi et al., 2006; Baba, 2006; Garba et al., 2010). Recently, serological evidence of Mokola and Lagos Bat viruses in Nigeria has also been reported (Dzikwi et al., 2010a, b; Nottidge et al., 2007).

The success of rabies control programs will depend on how much local dog epidemiology is known and taken into account when planning control strategies (Cleaveland et al., 2006; Hsu et al., 2003). This is because in most parts of Africa and Asia, rabies virus predominantly circulates within the dog population (Arai et al., 2001). Therefore, eradication of dog rabies is assumed to be the most logical solution to eliminate the risk of rabies to humans. There is a paucity of such information from Lagos state which is the most densely populated state in the country with both high human and dog populations. This study is therefore conducted to determine the prevalence of rabies antigen in brain of dogs slaughtered for consumption and those that died in veterinary clinics as well as to obtain retrospective data on dog bites/suspected dog rabies cases in Lagos State.

## 2. Materials and Methods

### 2.1 Study Area

The study was carried out in Lagos State located in southwest Nigeria between latitude 6°2´N and 6°4´N and between longitudes 2°45´E to 4°20´E. The State has a total population of about 9 million people (Census, 2006), and is the second most populated State in Nigeria. It is a costal State, bounded in the north and east by Ogun state, in the south by the Atlantic Ocean and in the west by the Republic of Benin and Togo. The sample frame included all military formations and veterinary clinics within the three senatorial districts of Lagos State. The three senatorial districts are Lagos West, Lagos East and Lagos Central senatorial districts. Lagos West has ten Local Government Areas (Ojo, Ifako Ijaye, Ikeja, Ajeromi Ifelodun, Mushin, Badagary, Amuwo Odofin, Oshodi Isolo, Alimosho and Agege) while Lagos East (Ikorudu, Epe, Kosofe, Shomolu and Ibeju Lekki) and Lagos Central (Etiosa, Apapa, Lagos Island, Lagos Mainland and Surulere) have five Local Government Areas each.

By random sampling, two military formations and veterinary clinics from two local government areas in Lagos West senatorial district (Wards B and E in Ajeromi Ifelodun local government area and wards A and E in Oshodi Isolo local government area) and one local government areas each from the Lagos East (ward C and E in Ibeju lekki local government area) and Lagos Central senatorial district (ward A and D in Etiosa local government area) were selected.

Brain samples were collected between February, 2011 and January, 2012 from dogs slaughtered in the “mammy markets” within the selected military formations by random sampling. Brain samples were also collected from all dogs that died (during the course of the study) in the veterinary clinics selected for the study.

### 2.2 Sample Collection

Following slaughter, a straw (5mm) or a 2ml disposable plastic pipette was introduced into the occipital foramen in the direction of an eye. The straw cut across the rachidian bulb, the base of the cerebellum, hippocampus, cortex and medulla oblongata (Barrat & Blancou, 1998; Bourhy & Sureau, 1991). The straw was then removed and the area containing the brain cut and deposited into a pre-labelled bijou plastic bottle and stored at -20°C. The samples were then transported to the Viral Zoonosis Laboratory of the Department of Veterinary Public Health and Preventive Medicine, Ahmadu Bello University Zaria where they were stored at -20°C until analyzed.

### 2.3 Direct Flourescent Antibody Technique (DFAT)

DFAT was performed in the Viral Zoonoses Laboratory of the Department of Veterinary Public Health and Preventive Medicine, ABU, Zaria on brain samples collected. The DFAT was performed as described by the Center for Disease Control and Prevention (CDC), Atlanta, USA (CDC, 2011) as follows;

Impression smear of the brain sample was prepared on a clean glass slide, air dried and fixed in cold acetone for 1hour at – 20°C.

The acetone fixed immersion smear of the sample was then stained with Flourescien-labelled anti-rabies immunoglobulin (FITC anti-rabies monoclonal globulin, Fujirebio Diagnostic, Inc. (FDI), USA).

The slides were incubated for 30 minutes at 37°C in a humid chamber and then washed with phosphate buffered saline (pH 8.5) three successive times over a period of 10 minutes.

Slides were air-dried after rinsing with distilled water. The slides were viewed at X 400 using a Fluorescent microscope (Meiji Techno Company Ltd, USA; Model mt6000 series).

### 2.4 Interpretation

The results was interpreted as positive if a bright apple-green fluorescence of particles ranging in size and morphology from “dust particles” to prominent cytoplasmic inclusion “Negri bodies” was observed under the microscope and negative when no specific apple-green fluorescence is exhibited under the fluorescent microscope i.e. rabies virus antigen is absent in all fields examined per impression.

### 2.5 Data Collection for Retrospective Studies

Retrospective data of total number of dogs vaccinated yearly and reported cases of dog bite and suspected rabies cases from January 2006 to December 2011 were collected from records of the veterinary hospitals in the three senatorial districts of Lagos State. Information on vaccination status, sex of offending dogs, quarantine decision and age of the victim were collected.

### 2.6 Data Analyses

The data obtained were presented using tables and Chi square or Fisher’s exact test was used where appropriate with the aid of SPSS version 17.0 was used to test for association of variables. The prevalence of rabies antigen in the brain of dogs sampled from slaughtered slabs and those that died in veterinary clinics was calculated using the formula.


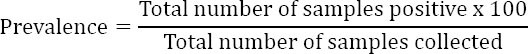


## 3. Results

### 3.1 Prevalence of Rabies Antigen among Dogs Slaughtered and those that Died in Clinics

A total of 444 brain samples; 401 (347 males and 54 females) from slaughter slabs and 43 (12 males and 31 females) from dogs that died in veterinary clinics were collected for this study. Only 7 (1.58%) out of the 444 brain samples collected and tested were positive for the rabies antigen using the DFAT. All the rabies positive brain samples were from slaughtered dogs (Table 1).

**Table 1:** Prevalence of rabies antigen in brain tissue of dogs in the three senatorial districts of Lagos State

Source of samples	No of samples tested			No. positive (%)
	Males	Females	Total	
Slaughter slabs	347	54	401	7 (1.75)
Clinics	12	31	43	0 (0.00)
**Total**	**359**	**85**	**444**	**7 (1.57)**

Chi square value 0.7626, *p value = 0.3825*

A total of 85 female and 359 were male dogs were sampled. Four out of the 7 positive dogs were males while the other 3 were females (Table 2).

**Table 2:** Sex based prevalence of rabies antigen in brain tissue of dogs in the three senatorial districts of Lagos State

Sex	Total no. sampled	No. positive (%)
Males	359	4 (1.11)
Females	85	3 (3.53)
**Total**	**444**	**7 (1.57)**

Fisher’s Exact Test *p value = 0.1320*

### 3.2 Data from Retrospective Studies

**Figure 1:**
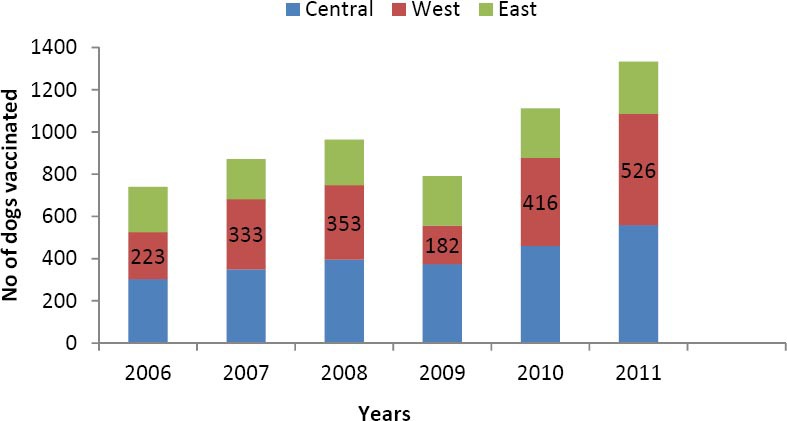
Number of dogs vaccinated each year against rabies from vaccination records of the Government and veterinary clinics in the three senatorial districts of Lagos state between 2006 and 2011

From the records of the veterinary clinics under the Lagos State Ministry of Agriculture and private veterinary clinics across the three senatorial districts, there was a yearly increase in number of dogs vaccinated from 740 in the year 2006 to 1333 in the year 2011 (Figure 1).

A total of 196 cases of dog bite were reported between 2006 and 2011 to the veterinary clinics (both government and private owned) in the three senatorial districts Lagos state. One hundred and fifty six of the offending dogs have been vaccinated against rabies while 40 have not been vaccinated. Majority (116) of the offending dogs were males, 76 were females while the sex of the remaining 4 dogs was not recorded. One hundred and eighty nine (96.43%) of the offending dogs were not quarantine because the bites were believed to have been “provoked” while 7 were quarantined. Out of the 7 dogs reportedly quarantined, six were released after the quarantine period while one died during the period. The dog that died was later confirmed positive for rabies by the National Veterinary Research Institute VOM, Nigeria. Majority (92) of the bite victims were males, 87 were females while the sex of 17 of the victims was not recorded. The victims (humans bitten by dogs) spread across all ages, but adults (109) were more affected compared to children (69) while the ages of 18 of the victims were not recorded (Table 3).

**Table 3:** Record of dog bite cases from 2006 – 2011 obtained from private and Government owned veterinary clinics in the three senatorial districts of Lagos state, Nigeria

Year	No. dog bite cases	Sex of offending dog			Vaccination status of dog		Was offending dog quarantined		Sex of victim			Age of victim		
		Male	Female	Unknown	Yes	No	Yes	No	Male	Female	Unknown	child	adult	unknown
2006	41	23	18	-	33	8	-	41*	20	13	8	13	20	8
2007	32	14	14	4	24	8	-	32*	16	11	5	12	14	6
2008	28	15	13	-	22	6	1	27*	9	17	2	7	19	2
2009	31	21	10	-	26	5	-	31*	16	15	-	13	18	-
2010	34	21	13	-	25	9	2	32*	13	19	2	11	21	2
2011	30	22	8	-	26	4	4**	26*	18	12	-	13	17	-
**Total**	**196**	**116**	**76**	**4**	**156**	**40**	**7**	**189**	**92**	**87**	**17**	**69**	**109**	**18**

* Dogs were not quarantined because bites were provoked

** dogs were quarantined and one died during the period (was confirmed rabies positive in National Veterinary Research Institute Vom

## 4. Discussion

The findings of this study showed that the rabies antigen is present in brain tissue of slaughtered dogs in Lagos State with a prevalence of 1.57%. This is in agreement with the findings of other workers (Aliyu et al., 2010; Garba et al., 2008, 2010). Domestic dogs have been reported to be the most common source of rabies infection to humans and other animals (John, 2005), with more than 95% of human cases caused by bites from rabid dogs (Ratsitorahina et al., 2007). Human exposure to dog bite is very common in this region (Dzikwi et al., 2010a). Therefore these slaughtered dogs pose a risk as potential sources of the rabies virus to other dogs, the dog owners and those that slaughter such dogs following bites or injury when processing such dogs for consumption. Some of the dogs slaughtered in Lagos State were reported to be sourced from other States. The presence of the rabies antigen in the brain tissue of these dogs is therefore, an indication of the likelihood of importation of the disease into the State since the dog trade and movement is unregulated.

There was a yearly increase in the number of vaccinated dogs, 223 in 2006 to 526 in 2011 (a total of 2033 vaccinations reported during the 6-year study period), however compared to the estimated dog population of 1,527,718 in Lagos state (Hambolu, 2013) this is very low. It is lower than the 70% total vaccination coverage recommended by the WHO (Coleman & Dye, 1996). This low vaccination level indicates that the populace may not be aware or do not consider the threat dogs pose in the epidemiology of rabies. It also indicates that a large proportion of dog population in Lagos state are at risk of been infected with the rabies virus.

The total number of dog bite cases/victims estimated in the three senatorial districts visited during the study period (2006-2011) was 196. Though the actual number may more than the records indicated, the number is low compared to the 247 cases reported by Bata et al. (2011) which they attributed to the high number of dogs in their study area i.e. the area (Kanke Local Government of Plateau State) is home the largest commercial dog market in West Africa. The absence of standard reporting makes accurate estimate of the exact incidence of dog bite injuries difficult, also some people do not report, seek medical treatment post – exposure especially when the wound is small. This is also complicated by the poor attitude of record keeping in some of the veterinary clinics as observed during the study.

The epidemiological profile of offending dogs showed that about 79.59% (196) and 20.41% (40) were vaccinated and unvaccinated against rabies respectively. This is in contrast to the findings of Bata *et al*. (2011) who reported that about 82.17% of the offending dogs were unvaccinated. Majority (96.43%) of the offending dogs were not vaccinated because the bites where believed to be “provoked”. Though, majority of the dogs were reported to be vaccinated reports of presence of rabies antigen in the brain of apparently healthy dogs (vaccinated and unvaccinated) as well as shedding of the virus by such dogs has been made. Therefore, such bites may still pose a potential threat in the transmission of the virus if the dogs are shedding the virus.

Both sexes of dogs were responsible for bite cases. The aggressive behavioural change of male dogs during mating and breeding season can also contribute for biting. Similarly, nursing bitches are naturally aggressive and can bite humans under normal condition to protect the newly born puppies from some individuals/intruders (Deressa et al., 2010). About 47% of the bite victims were males compared to females (44.90%), this could be explained due to the fact that men are more likely to go out of their homes for work as compared to women. This finding is also in agreement with the findings of (Aghahowa & Ogevoen, 2010) in Benin city, however, contrary to the findings of Aghahowa & Ogbevoen (2010); Deressa et al. (2010); Bata et al. (2011), adult (55.61%) compared to children (35.20%) were the major bite victims. Generally dogs can be aggressive when provoked. Other victims can be strange visitors who are visiting for the first time or neighbours and family members of dog owners.

## 5. Conclusion

The findings of this study showed rabies antigen was present in the brain tissue of dogs slaughtered for consumption in Lagos State with a prevalence of 1.57% indicating these dogs may serve as potential reservoir and source of spread of the virus to the public especially the dog owners, processors and consumers. Despite the implication and severity of reported rabies cases in Nigeria basic control measures such as vaccinating dogs against the diseases is still very inadequate. There is a poor attitude towards record keeping especially as it relates to dog bites/suspected rabies cases among veterinary practitioners both in the government and private clinics across Lagos State. There is need to enlighten the populace on the need to report dog bite cases so that proper medical attention can be received. There is also need for government owned veterinary clinics to improve on record keeping especially as it relates to dog bites/rabies.
